# Haploidentical CD7 CAR T-cells induced remission in a patient with *TP53* mutated relapsed and refractory early T-cell precursor lymphoblastic leukemia/lymphoma

**DOI:** 10.1186/s40364-022-00352-w

**Published:** 2022-02-07

**Authors:** Hai-ping Dai, Wei Cui, Qing-ya Cui, Wen-juan Zhu, Hui-min Meng, Min-qing Zhu, Xia-ming Zhu, Lin Yang, De-pei Wu, Xiao-wen Tang

**Affiliations:** 1 National Clinical Research Center for Hematologic Diseases, Jiangsu Institute of Hematology, The First Affiliated Hospital of Soochow University, Suzhou, 215006 China; 2 Institute of Blood and Marrow Transplantation, Collaborative Innovation Center of Hematology, Soochow University, Suzhou, 215123 China; 3 PersonGen BioTherapeutics (Suzhou) Co., Ltd., Suzhou, 215123 China; 4 The Cyrus Tang Hematology Center, Soochow University, Suzhou, 215123 China; 5 Department of Hematology, the First Affiliated Hospital of Soochow University, Jiangsu Institute of Hematology, Suzhou, 215006 China

**Keywords:** Chimeric antigen receptor T-cells, CD7, Early T-cell precursor lymphoblastic leukemia/lymphoma, Relapsed / refractory

## Abstract

Patients with relapsed/refractory early T-cell precursor lymphoblastic leukemia/lymphoma (ETP-ALL/LBL) respond poorly to traditional therapy and have dismal prognosis. CD7 is a promising therapeutic targets for chimeric antigen receptor modified T cell therapy (CART) due to its widely expression in almost all T-cell malignancies. Here we present the anti-CD7 CART therapy in a 11-year-old male with *TP53* mutated relapsed/refractory ETP-ALL/LBL. The patient suffered second relapse after haploidentical hematopoietic stem cell transplantation, showing resistance to 4 lines salvage therapies including venetoclax. Nanobody derived CD7-CART cells were manufactured by co-transducing CAR-T cells with a CD7 protein expression blocker. 70.5% of blasts (CD7 expression: 92.6%) and extensive extramedullary disease (mediastinal mass, enlarged lymph nodes and spleen) were observed prior to CD7-CART-cell therapy. A total of 5 × 10^6^/kg donor-derived CD7-CART-cells were infused. Hematological and extramedullary remission were both achieved, with persistence of CD7-CART-cells be detected until the last followup at 96th days after the infusion. Reversible adverse effects including grade 3 cytokine release syndrome and macrophage activation syndrome were observed. This case demonstrated that CD7-CART was a potent and safe salvage therapy in relapsed/refractory ETP-ALL/LBL patient with high tumor burden.

**Trial registration:** ClinicalTrials. gov, NCT04785833, Registered on March 8, 2021, prospectively registered.

**Supplementary Information:**

The online version contains supplementary material available at 10.1186/s40364-022-00352-w.

**To the editor**:

Early T cell precursor lymphoblastic leukemia/lymphoma (ETP-ALL/LBL) shows higher remission failure/relapse rates and worse outcomes compared with other T-ALL subtypes [1, 2]. CAR T-cell therapy is a promising salvage strategy in T-ALL, however, it has not been independently reported in ETP-ALL/LBL patients. Manufacturing difficulty resulted from shared expression of antigens between normal and malignant T-cells poses the main challenge [3]. We constructed the first CD7 CAR-modified NK cell lines based on anti-CD7 nanobody sequences [4] and demonstrated its robust anti-tumor activity against malignant T cells in vitro. Base on this, we developed non gene-editing CD7 CAR-T cells which overcome the fratricide of CD7 CAR-T cells through preventing expression of CD7 in the cell membrane with a protein expression blocker [5] (Fig. 1a, Supplementary Fig. 1–2). Here, we report the successful application of this anti-CD7 CAR-T cell product in a relapsed/refractory ETP-ALL/LBL patient.

**Fig. 1:**
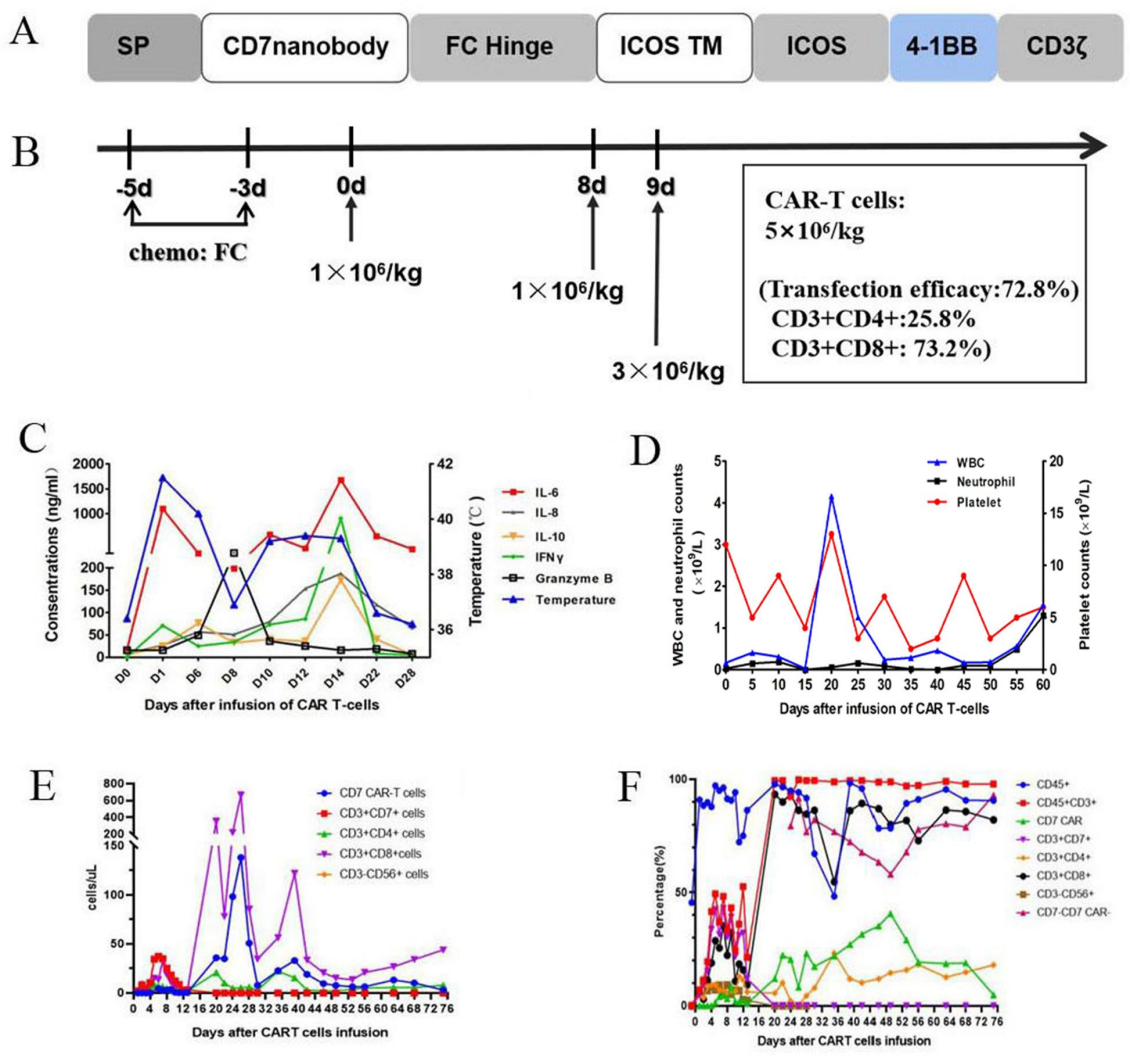
Characteristics of CD7 CAR T-cells (**a**, **b**) and change of cytokines and amplification of CAR T-cells (**c**-**f**). **a**, Schematic structure of the CD7 CAR T-cells; **b**, CAR-T cells were infused at a total of 5 × 10^6^/kg for 3 days; **c** Change of cytokines and temperature in the first month after CD7 CAR T-cells infusion. **d** Change of blood cell counts after CD7 CAR T-cells infusion. **e**, **f** Flowcytometry analysis of absolute (**e**) and relative CAR T-cell copies and the fraction of T-cells (**f**) in the peripheral blood samples after CAR T-cells infusion

An 11-year-old male was diagnosed with ETP-ALL/LBL in February 2016. He underwent haploidentical hematopoietic stem cell transplantation at CR2 in January 2019. The disease relapsed again in January 2021. After failure of 4 lines of salvage therapies (venetoclax with decitabine, high-dose cytarabine-based chemotherapy, chidamide and donor-derived CD38 CAR-T cell therapy), he was enrolled in an anti-CD7 CAR-T clinical trial (NCT04785833). Before infusion of anti-CD7 CAR-T cells, bone marrow (BM) showed 70.5% of blasts (Fig. 2a). A complex karyotype with *ETV6*, *NOTCH1* and *TP53* mutations were detected. Only 1.5% of donor cells were detected in the BM (Fig. 2c). Flow cytometry revealed 58.5% of blasts (positive for cCD3/CD7/CD15/CD33/CD34; weakly positive for CD5 and negative for CD1a/CD8). 92.6% of blasts were positive for CD7 (Fig. 2d) (Supplementary Table 1). PET-CT scan revealed extensive extramedullary involvement, including a mediastinal mass (5.0 cm × 5.7 cm × 4.7 cm) and high FDG metabolism in the spleen and nasopharyngeal, cervical, mediastinal, abdominal, and inguinal lymph nodes (Fig. 2f). Chemotherapy (fludarabine 30 mg/m^2^ and cyclophosphamide 300 mg/m^2^) was administered 5, 4, and 3 days before the first infusion of HSCT donor-derived CAR-T cells (April 15, 2021), followed by two once-daily infusions at 8 and 9 days after the first infusion. The effective anti-CD7 CAR-T cells totaled 5 × 10^6^ cells/kg (Fig. 1b).

**Fig. 2:**
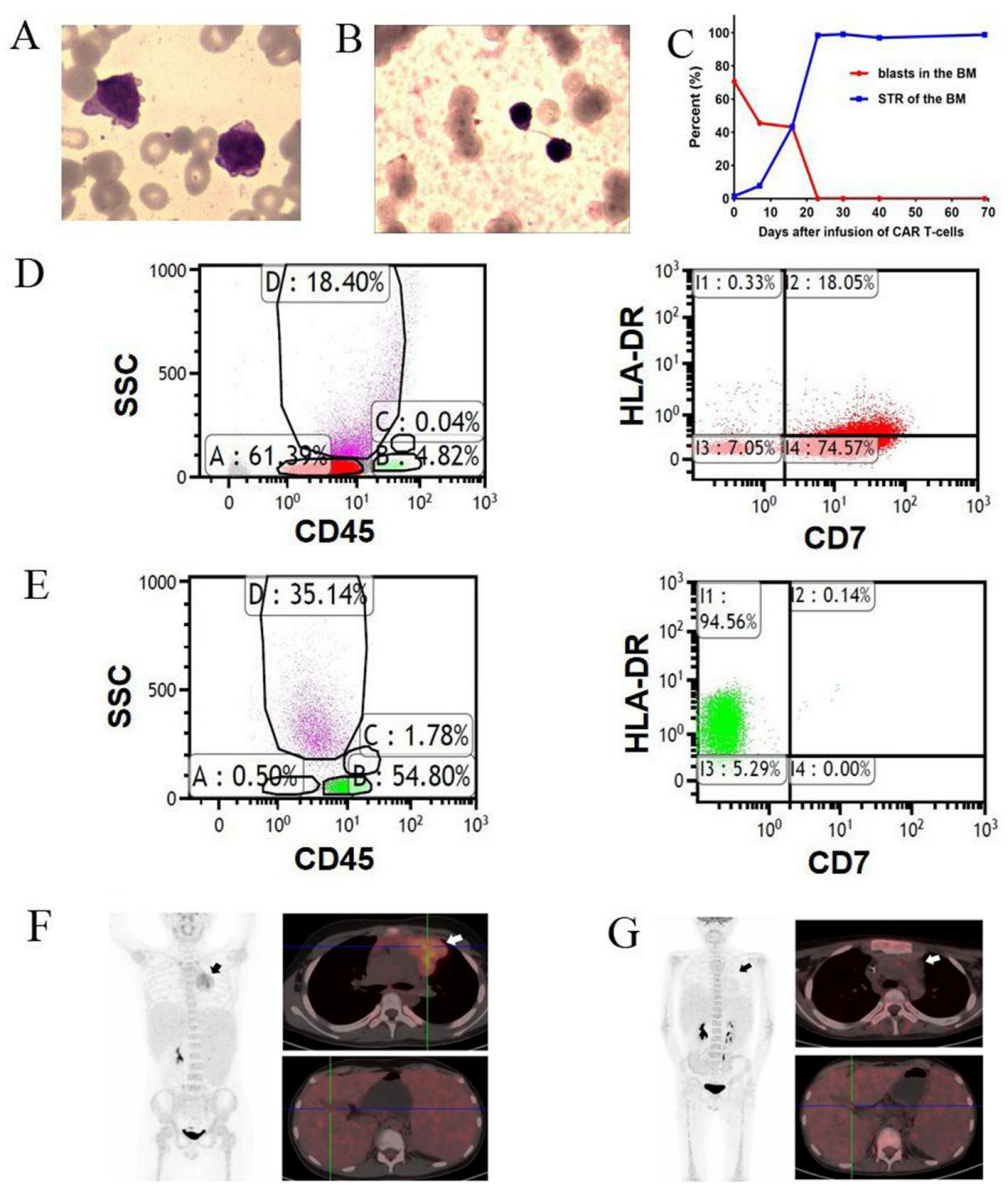
BM analysis and PET-CT scan of the patient before and after CD7 CAR T-cells infusion. **a**, BM morphology before infusion of  CD7 CAR T-cells; **b**, BM morphology after infusion of CD7 CAR T-cells; **c**, Change of percentage of BM blasts and donor chimerism (STR) before and after CD7 CAR T-cells infusion; **d**, **e**, Flow cytometry analysis before (**d**) and after (**e**) CD7 CAR Tcells infusion. **f**, **g** PET-CT scan before (**f**) and after (**g**) CD7 CAR T-cells infusion

The patient developed a high fever (39.6 °C, peaked at 41.1 °C the next day and lasted for 15 days) and tachycardia approximately 24 h after the first infusion (Fig. 1c). The peaks of serum IL-6 (93 times higher than baseline) and IFNγ were detected on the 14th day postinfusion (Fig. 1c). Pancytopenia, hypotension and pleural effusion were observed, with no signs of organ toxicity or immune effector cell-associated neurotoxicity syndrome. Low fibrinogen, elevated ferritin, NK cell deficiency and elevated soluble CD25 were observed. Grade 3 cytokine release syndrome and macrophage activation syndrome were considered as described [6, 7] and were relieved with tocilizumab, dexamethasone, plasma exchange and supportive care. White blood cells and neutrophils returned to normal on the 57th day, and independence of red blood cell infusion was achieved on the 50th day after infusion (Fig. 1d). Platelets were still dependent on transfusion at the last follow-up. No activation of CMV or EBV and no signs of GVHD were observed.

The BM aspirates showed hypoplasia with no blasts according to morphology and flow cytometry, with full donor chimerism 30 days after CAR T-cells infusion (Fig. 2b, c, e). BM aspirates were normocellular with no blasts, 4.4 × 10^− 4^ of blasts by flow cytometry, showed normal karyotype and full donor chimerism and were TP53 mutation-negative on day 91 (Supplementary Table 1). PET-CT scan at day 100 showed disappearance of the mediastinal mass and enlarged lymph nodes with no hypermetabolic lesions in other lymph nodes or the spleen (Fig. 2g). The CAR-T cells remained detectable, with no CD7-positive T cells and CD7-negative T cells as the predominant CD3-positive population (62–92%) in the PB at the last follow-up (Fig. 1e-f, Supplementary Fig. 3–4).

ALL/LBL exhibits universal overexpression of T-cell markers such as CD4, CD5 and CD7 [8]. CD4- and CD5-CAR-T cells were only evaluated in preclinical studies [9, 10]. Autologous CD7 CAR-T cell therapy was reported in a relapsed pediatric T-ALL [11]. HSCT donor-derived CD7 CAR-T cell therapy was reported in 12 T-ALL cases [12]. Compared with those patients, this is the first ETP-ALL/LBL case, who had a significantly higher tumor burden (70.5% of blasts in the BM and extensive extramedullary infiltration) before CAR-T cells infusion. Our patient achieved deep remission after the CD7 CAR-T cell therapy though he had unfavorable genetics and was resistant to all the available salvage treatments. This encouraging results not only confirmed our in vitro assays (Supplementary Fig. 2), but also also implied that this nanobody-based CD7 CAR-T cells could be a promising strategy for relapsed/refractory ETP-ALL/LBL.

## Supplementary Information

**Additional file 1 **: **Supplementary Figure 1**. Flow cytometry analysis on the anti-CD7 CAR T-cells. **Supplementary Figure 2**. Cytotoxicity analysis of the anti-CD7 CAR T-cells. **Supplementary Figure 3**. Timeline of treatments and responses. **Supplementary Figure 4**. T-cell fractions in the PB post CAR T-cells infusion. **Supplementary Table 1**. Baseline clinical characteristics of the patient. **Supplementary Table 2**. A panel of 222 genes detected by next generation sequencing.

## Data Availability

The datasets supporting the conclusions are included within this article.

## Abbreviations

BM: Bone marrow
CAR: Chimeric antigen receptor
CR: Complete remission
CRS: Cytokine release syndrome
ETP-ALL/LBL: Early T-cell precursor lymphoblastic leukemia/lymphoma
HSCT: Hematopoietic stem cell transplantation
