# Supplementary material for: Haploidentical CD7 CAR T-cells induced remission in a patient with TP53 mutated relapsed and refractory early T-cell precursor lymphoblastic leukemia/lymphoma
Source: Biomark Res. 2022 Feb 7;10:6. doi: 10.1186/s40364-022-00352-w (PMC8822664; doi:10.1186/s40364-022-00352-w)
Supplement: Supplementary file 1 — Additional file 1 : Supplementary Figure 1. Flow cytometry analysis on the anti-CD7 CAR T-cells. Supplementary Figure 2. Cytotoxicity analysis of the anti-CD7 CAR T-cells. Supplementary Figure 3. Timeline of treatments and responses. Supplementary Figure 4. T-cell fractions in the PB post CAR T-cells infusion. Supplementary Table 1. Baseline clinical characteristics of the patient. Supplementary Table 2. A panel of 222 genes detected by next generation sequencing. [file 40364_2022_352_MOESM1_ESM.pdf]

Supplementary figures

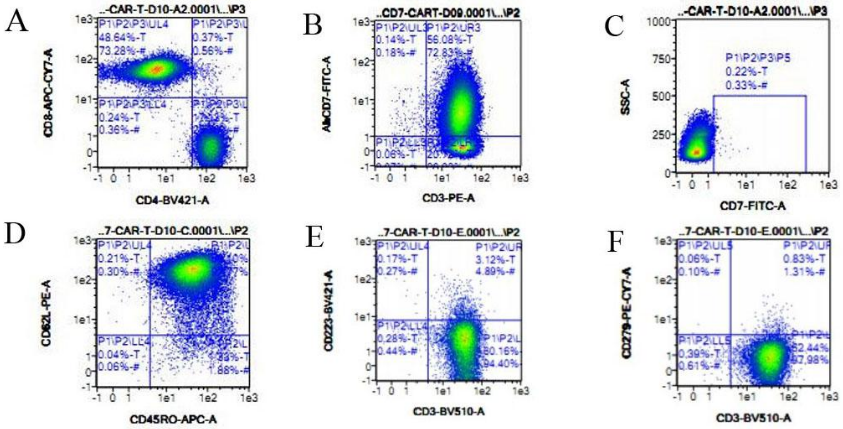

Supplementary figure 1. a, CD4 and CD8 expression of the CD7 CAR T-cells detected with flowcytometry; **b**, CD7-CAR expression of the CD7 CAR T-cells detected with flowcytometry; **c**, CD7 expression of the CD7 CAR T-cells detected with flowcytometry; **d-f**, CD62L, CD223 and CD279 expression of the CD7 CAR T-cells detected with flowcytometry.

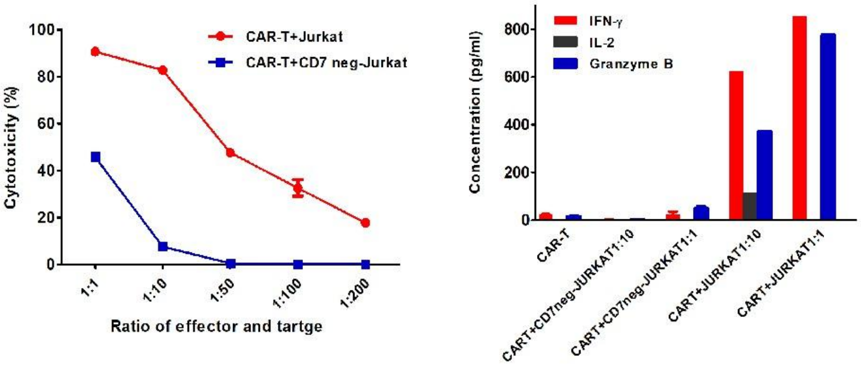

Supplementary figure 2. a, Cytotoxicity analysis of CD7 CAR T-cells at different effector and target ratios. b, Concentrations of cytokines secreted by CAR T-cells.

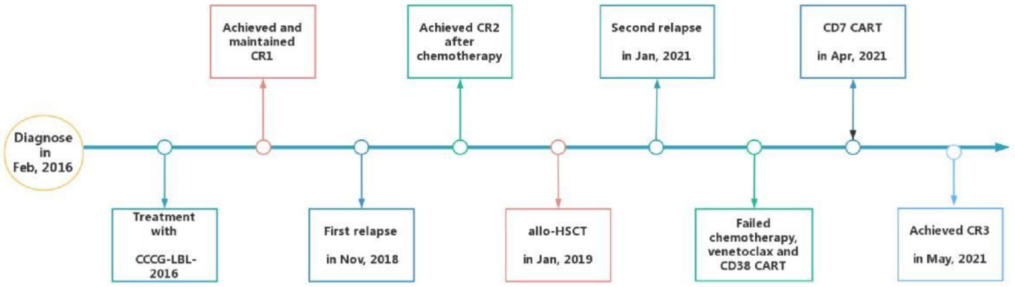

Supplementary figure 3. Time line and treatment response of the patient.

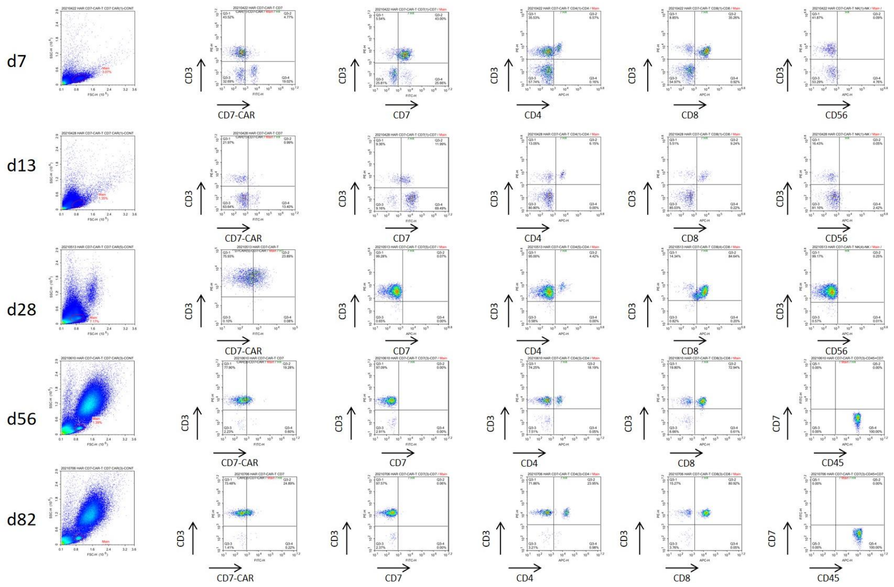

Supplementary figure 4. T-cell fractions in the PB post CAR T-cells infusion. The results showed that the CD7 expression of T-cells were negative since day 28.

Supplementary Table 1. Baseline characteristics of the patient

Age /Sex (yr)						Disease Status before CD7 CAR T-cels infusion								Disease Status on day 91 after CD7 CAR T-cels infusion					
	Prior Therapies					Disease Burden			Genetic Aberrations			Immunophenotype		Disease Burden					Chimerism Status
	Lines (no.)	1st Line	2nd Line	3rd Line	4th Line	BM Blasts (% by morphology)	BM Blasts (% by flow cytometry)	EMD	Fusion Gene	Somatic Gene Mutation	Complex Karyotype	(Positive expression,or dim/partial expression as indicated)	CD7 Expression (%)	BM Blasts (% by morphology) at day 91	BM Blasts (% by flow cytometry) at day 91	EMD at day 100	Somatic Gene Mutation	Karyotype	
13/ M	4	CCCG-LBL-2016	allo-HSCT	Chemo+ venetoclax chidamide	CD38 CART	70.5	58.5	mediastinal mass +lymph node +spleen	No	*ETV6 p.Ile48fs , NOTCH1 p.Phe1606delinsLeuPro, TP53 p.Tyr234Cy*	# Yes	*CD2, cCD3,CD5 ^{dim}, CD7, CD11b, CD15, CD33, CD34, CD38, CD56, CD99 ^{dim}*	92.3	0	< 4.4×10^{-4}	CR	Negative	46,XY[13]	99%

46，XY, del(5)(q23q35), t(12;14)(q13;q32)[2]/45,idem,-16[3]/46,idem, ?der(16)t(16;?)(p12;?)[1]/45,idem, del(3)(q23q26),-4, der(16)t(16;?)(p12;?)[3]/46,XY[1]

Supplementary Table 2. A pannel of 222 genes detected by next generation sequencing

ABL1	CD274	EPHA7	JAK3	PIM1	SRSF2
ANKRD26	CD28	EPOR	KANSL1	PLCG1	STAG2
APC	CD58	ERBB3	KDM6A	PLCG2	STAT1
ARAF	CD79A	ETNK1	KIT	POT1	STAT2
ARC	CD79B	ETV6	KLF2	PPM1D	STAT3
ARID1A	CDKN1A	EZH2	KMT2A	PRDM1	STAT4
ARID1B	CDKN2A	FAS	KMT2C	PRKCB	STAT5A
ARID2	CDKN2B	FAT1	KMT2D	PTEN	STAT5B
ARID5B	CDKN2C	FBXW7	KRAS	PTPN1	STAT6
ASXL1	CEBPA	FGFR1	MAP2K1	PTPN11	SUZ12
ASXL2	CEBPE	FGFR3	MAPK1	PTPRD	SYK
ASXL3	CHD8	FLT3	MECOM	PTPRK	TBL1XR1
ATG5	CIITA	FOXO1	MED12	RAD21	TCF3
ATG2B	CRBN	FOXO3	MEF2B	RAF1	FAM46C
ATM	CREBBP	FYN	MET	RB1	TET1
ATRX	CRLF2	GATA1	MFHAS1	RELN	TET2
B2M	CSF1R	GATA2	MGA	RET	TNFAIP3
BCL2	CSF3R	GATA3	MPL	RHOA	TNFRSF14
BCL6	CSMD1	GNA13	MUM1	RPS15	TNFRSF1B
BCOR	CTCF	GNAI2	MYC	RRAGC	TP53
BCORL1	CTLA4	HACE1	MYD88	RUNX1	TP63
BIRC3	CTNNB1	HNRNPA2B1	NF1	SAMHD1	TP73
BLNK	CUL4B	HRAS	NFKB1	SETBP1	TRAF3
BRAF	CXCR4	ID3	NFKB2	SETD2	TYK2
BRCA1	CYLD	IDH1	NFKBIE	SF3B1	U2AF1
BRCA2	DDX3X	IDH2	NOTCH1	SGK1	UNC13D
BRCC3	DDX41	IGLL5	NOTCH2	SH2B3	VAV1
BTG1	DHX15	IKZF1	NPM1	SH2D1A	VHL
BTK	DIS3	IKZF2	NRAS	SMARCA4	VPREB1
CALR	DNM2	IKZF3	NT5C2	SMARCB1	WT1
CARD11	DNMT3A	IL7R	PAX5	SMC1A	XPO1
CBL	ECT2L	INO80	PDCD1LG2	SMC2	ZAP70
CCND1	EED	INPP5D	PDGFRA	SMC3	ZBTB7A
CCND2	EGFR	IRF4	PDGFRB	SOCS1	ZEB2
CCND3	EGR1	ITPKB	PHF6	SOS1	ZMYM3
CCR4	EGR2	JAK1	PIGA	SPEN	ZNF292
CCR7	EP300	JAK2	PIK3CA	SRP72	ZRSR2
